# Supplementary material for: Larger rock extraction sites could improve the efficiency of enhanced rock weathering in the United Kingdom
Source: Commun Earth Environ. 2025 Aug 15;6(1):666. doi: 10.1038/s43247-025-02656-9 (PMC12356697; doi:10.1038/s43247-025-02656-9)
Supplement: Supplementary file 2 — Supplementary Information 1 [file 43247_2025_2656_MOESM2_ESM.pdf]

Supplementary Information

Spatio-temporal allocation within the rock supply chain influences the efficiency of enhanced rock weathering deployment at scale

Mohammad Madankan[1], Euripides Kantzas[2], Rafael M. Eufrasio[3], Sylvia H. Vetter[4], Lenny Koh[3], Pete Smith[4], David J. Beerling[2], Phil Renforth[1*]

[1] School of Engineering and Physical Sciences, Heriot-Watt University, Edinburgh Campus, Edinburgh, UK

[2] Leverhulme Centre for Climate Change Mitigation, Department of Animal and Plant Sciences, University of Sheffield, Sheffield, UK

[3] Advanced Resource Efficiency Centre, Management School, University of Sheffield, Sheffield, UK

[4] Institute of Biological and Environmental Sciences, University of Aberdeen, Aberdeen, UK

Supplementary Figures

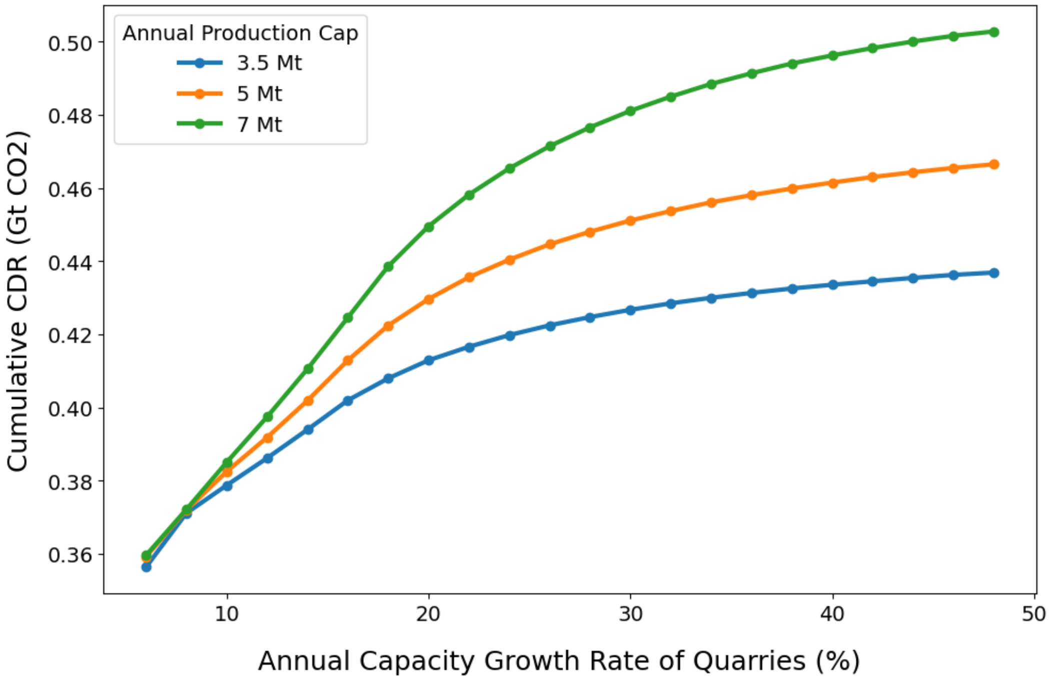

Fig. S1 Scenarios of varying capacity growth rate and annual production cap and their impact on the total CDR. This demonstrates that both the annual capacity growth rate and the maximum production cap of quarries are critical factors influencing the final CDR achieved through ERW. Higher growth rates and larger production caps enable the model optimise the quarries contribution to rock supply, thereby maximising the cumulative CDR.

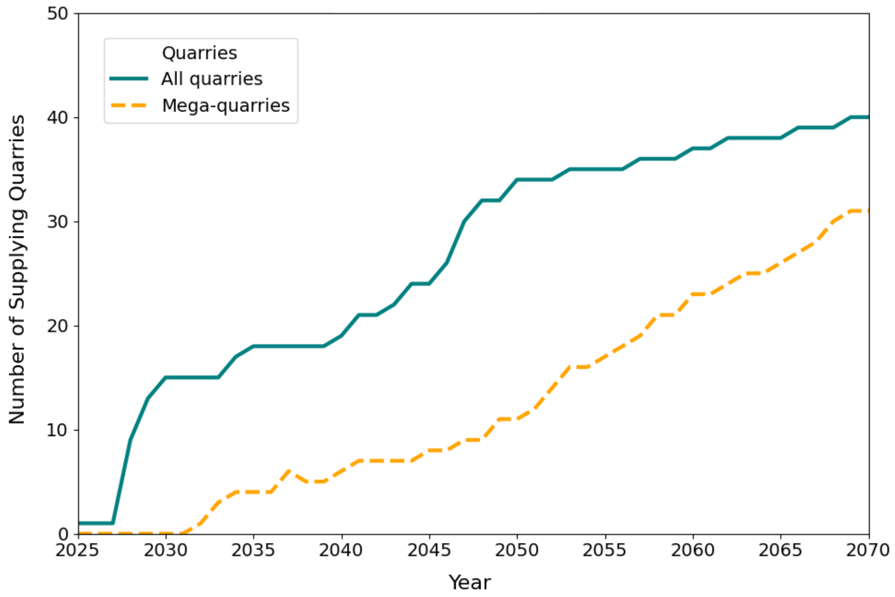

Fig. S2 Projected total number of required quarries for ERW and the number of Mega-quarries with an annual production of over 1 Mt over time. This projection is based on a scenario that relies solely on the expansion of currently active quarries, with a production cap of 3.5 Mt yr-1. This illustrates the temporal evolution of the number of supplying quarries in order to meet the increasing demand for rock supply under the modelled scenarios (S1, S2, and S3). The solid teal line represents the total number of quarries supplying rock, while the dashed orange line represents the subset of these quarries that are

classified as "Mega-quarries," defined as quarries with an annual production capacity exceeding 1 Mt. As shown in this figure, relying only on expansion of existing active quarries for fulfilling the target demand requires over 30 mega-quarries (quarries with over 1 Mt yr-1 capacity) to operate by 2070.

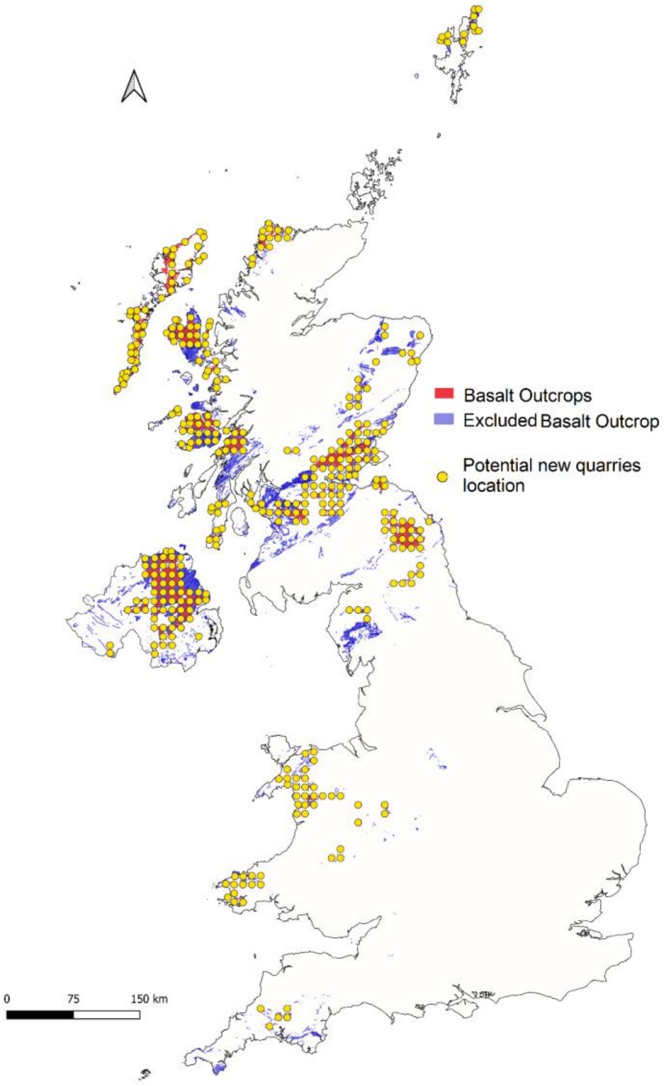

Figure S3 Map of basalt outcrops suitable for ERW in the UK. The blue regions shows the basalt outcrops that are excluded for potential location of new quarries as they overlap with either protected or urban areas. The yellow dots represent the potential location of new basalt quarries for ERW. This shows the potential locations for new quarries that are suitable for ERW. Basalt outcrops (red areas) are the regions where suitable rock material can be sourced for ERW. Excluded basalt outcrops (blue areas) while also containing basalt, have been excluded from consideration due to being located in protected areas, urban regions, or other land uses that are not suitable for quarrying activities. The exclusion was based on overlaying the geological map with maps of the UK's protected areas and urban regions, ensuring that quarrying activities do not conflict with conservation or land use priorities. The yellow dots represent the centroids of 10x10 km grid cells that were identified as potential new quarry sites. These locations were determined by first extracting suitable geological units from the geological map and then filtering out unsuitable areas (protected areas, urban regions, etc.). The remaining suitable areas were then converted into a grid, with the centroids of these grids being

visualised as the potential approximate locations for opening new quarries if required for scalability of ERW.

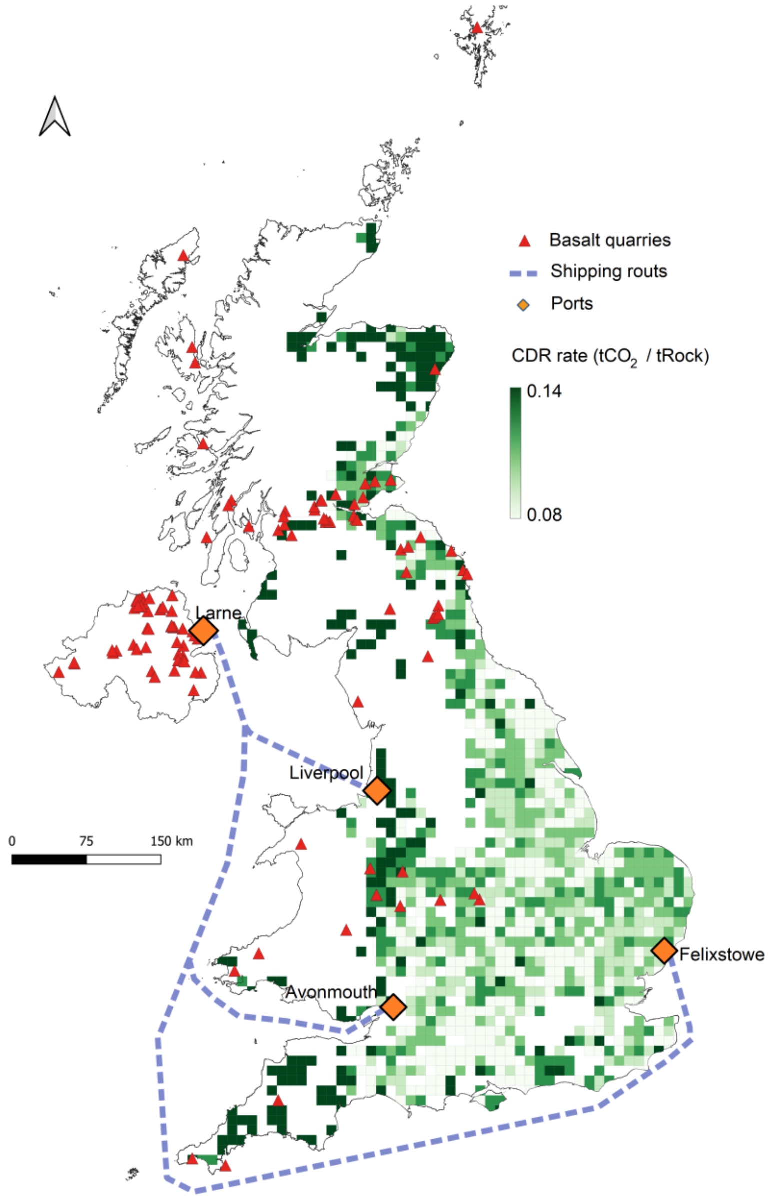

Figure S4 Map of ports and potential shipping routes in the UK for transporting rock from Northern Ireland to England for ERW. The grid cells represent croplands where basalt can be applied, with the colour map indicating the potential CDR rate achievable in each cropland. This illustrates three proposed shipping routes connecting one port in Northern Ireland (Larne) to three key ports in England (Liverpool, Avonmouth, and Felixstowe) for the transport of basalt rock for ERW. These ports were selected based on their strategic proximity to croplands in England with high CDR potential, as indicated by the color-coded grid cells. Shipping by sea is proposed as a low-carbon alternative to road transport, minimising the CO_2 footprint of long-distance logistics. While croplands in Scotland can be supplied by quarries within Scotland, croplands in southern and southeastern England lack nearby quarry resources. Therefore, shipping basalt from Northern Ireland to the selected ports provides an efficient solution to supply these croplands, enabling effective ERW deployment in regions distant from primary rock sources.

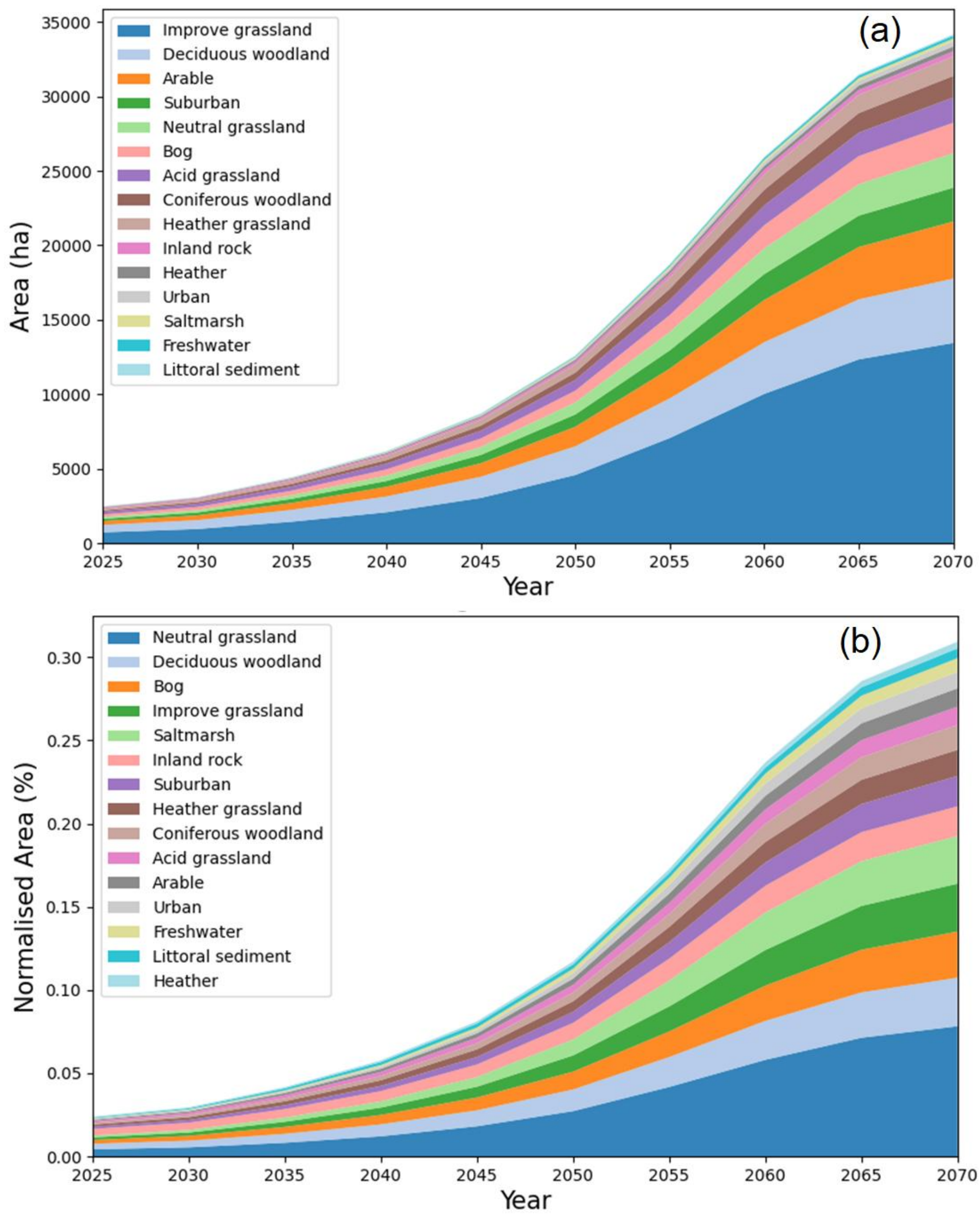

Figure S5 Projected land-use conversion under ERW quarry expansion (Scenario S3.a, 2025–2070). (a) Cumulative area (ha) of each land-use class changed to quarrying. (b) expresses these land use changes as a percentage of each land-use type's total UK extent. (see supplementary text 1 for further explanation).

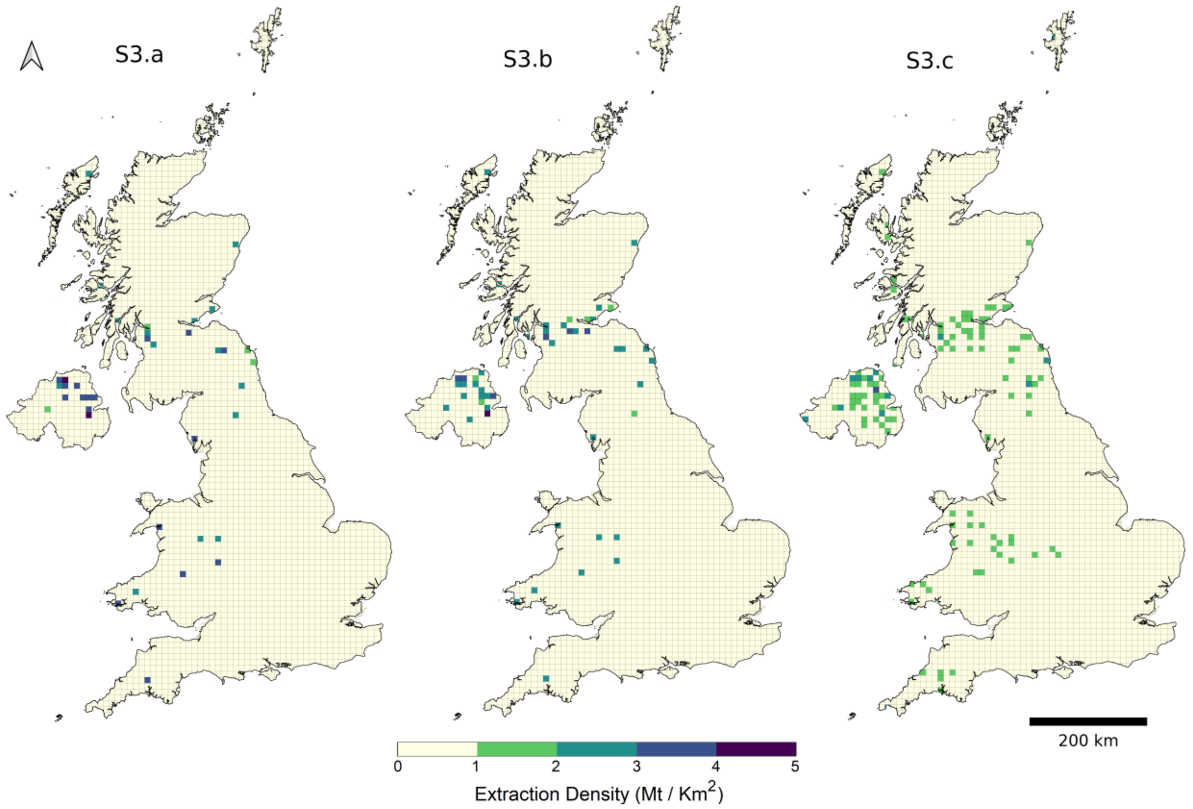

Figure S6 Extraction density grided map (10 x 10 km) representing the cumulative reserve requirement divided by the area, illustrating the potential concentrated environmental and social impact of extraction activities. This map shows the cumulative rock to be extracted per unit area (Mt km^{-2}) over 2025–2070 in S3 sub scenarios. In the high-supply, active-only scenario (S3.a), extraction is intensely localised, with a few grid cells exceeding 4 Mt km^{-2}. Imposing a lower production cap of 2 Mt yr^{-1} per quarry (S3.b) spreads production across more quarries, reducing peak densities to around 3 Mt km^{-2} and generating a broader band of moderate densities (2–3 Mt km^{-2}). A further reduction to a 1 Mt yr^{-1} cap (S3.c) diffuses extraction pressure still more, yielding mostly low densities (1–2 Mt km^{-2}) across the grid. This grid-based view complements the county-scale extraction-density map (Fig. 4).

Supplementary Tables

Table. S1: Input CO_2 footprint data of different ERW processes considered in the spatio-temporal allocation model.

**Process**	**CO_2 emission**	**Reference**
Rock extraction	25 kWh t^{-1} Rock	Kantzas et al., 2022 [1]<br>Brown et al., 2010 [2]
Rock crushing & grinding to 10 μm	112 kWh t^{-1} Rock	Renforth; 2012 [3]
Road Transport	50 grCO2 t.km $^{-1}$	Kantzas et al., 2022 [1]
Bulk career	6.49 grCO2 t.km $^{-1}$ *	Foteinis et al., 2022 [4]
Rock dust spreading	21 kWh t^{-1} Rock	Renforth, 2012 [3]

*Estimated based on IPCC 2021 GWP10050 for a 50 kt bulk carrier for dry goods, which indirectly includes some empty trips throughout its 25 years lifetime.

Table. S2: Life cycle emission of electricity in the UK by 2070**;** data from (Euripides et. al., 2022)

**Year**	**LCE (grCO2/kWh)**	**Year**	**LCE (grCO2/kWh)**
2025	177.5	2048	-49.7
2026	113.3	2049	-51.1
2027	79.0	2050	-53.2
2028	57.6	2051	-55.1
2029	49.3	2052	-57.2
2030	39.6	2053	-59.3
2031	32.0	2054	-61.5
2032	23.1	2055	-63.5
2033	16.2	2056	-65.4
2034	9.7	2057	-67.4
2035	5.4	2058	-67.8
2036	-2.8	2059	-68.0
2037	-9.0	2060	-68.1
2038	-17.2	2061	-68.0
2039	-23.5	2062	-68.2
2040	-32.0	2063	-67.9
2041	-38.3	2064	-67.4
2042	-32.7	2065	-66.8
2043	-33.5	2066	-66.3
2044	-43.4	2067	-65.8
2045	-44.3	2068	-65.3
2046	-46.3	2069	-64.8
2047	-47.5	2070	-64.6

Table. S3 Dedicated shipping routes, distances, and corresponding CO_2 emissions for transporting rocks from Northern Ireland to England (port-to-port).

**Rout**	**Distance NM**	**Emission (kgCO2 / tRock)**
Port Larne – Liverpool Port	143	1.7
Port Larne – Avonmouth Port	326	3.9
Port Larne – Felixstowe Port	669	8.0

*Nautical Mile is equal to 1.852 km.

Supplementary Note 1

To estimate future land use impacts under our ERW up-scaling scenarios, we began with the 70 active quarries in our UK inventory, each represented by its current extraction boundary and annual production. For each quarry, we computed an area-to-production ratio (m^2 per t yr^{-1}), on average about **1.1 m^2 per t yr^{-1},** by dividing its mapped footprint by its reported annual rock production. Using scenario S3.a as an example, at each five-year step we multiplied each quarry's projected increase in annual extraction by the area-to-production ratio to compute the additional land area required over that interval. In QGIS, we generated site-specific buffer polygons matching those expansion areas around each quarries existing boundary. Overlaying these projected expansion footprints onto the UK's Land Cover map [5,6] allowed us to quantify the forecasted area of each land-use class (e.g., arable, grassland etc.) that would be converted to quarrying over time.

We note that our expansion polygons are generated as circular buffers around each quarry boundary to match the estimated additional area. This simplification does not capture the likely directional growth constrained by existing infrastructure (roads, rail lines), topography, or planning constraints. Our circular buffers therefore serve only to approximate the potential land-use change for each site.

Fig. S5.a presents the cumulative projected area of each land-use class expected to be converted to quarrying between 2025 and 2070. To illustrate the significance of these projections, we also express them as a percentage of each class's total UK extent, thereby identifying which land use face the greatest proportional impact over time as shown in Fig. S5.b.

Under our most ambitious ERW-upscaling scenario (S3), the total land converted to quarry footprints rises from just a few hundred hectares in 2025 to roughly 33,000 ha by 2070 (Fig. S5.a). The largest absolute changes occur in improved grassland, deciduous woodland and arable land, together accounting for about two third of total projected land use change.

When viewed as a share of each land-use type (Fig. S5.b), even the most impacted land use, improved grassland, sees only about 0.08 % of its UK extent converted by 2070. Deciduous woodland and arable land each lose under 0.06 % of their total area, while all other classes remain below 0.03 %.

These projections show that ERW-driven quarry expansion will convert tens of thousands of hectares over 50 years but, spread across all land use types, the proportional impact on any single land-use category remains below 0.1 %. This suggests that, although absolute land-take is non-negligible, its relative footprint is modest compared to the total extent of UK land uses.

Supplementary References:

1. Kantzas, E. P. et al. Substantial carbon drawdown potential from enhanced rock weathering in the United Kingdom. Nat. Geosci. 15, 382–389 (2022). https://doi.org/10.1038/s41561-022-00925-2
2. Brown, T. J., Coggan, J. S., Evans, D. J., Foster, P. J., Hewitt, J., Kruyswijk, J. B., Millar, D. L., Smith, N. & Steadman, E. J. Underground Mining of Aggregates: Main Report. British Geological Survey (2010).
3. Renforth, P. The potential of enhanced weathering in the UK. Int. J. Greenhouse Gas Control 10, 229–243 (2012). https://doi.org/10.1016/j.ijggc.2012.06.011
4. Foteinis, S., Andresen, J., Campo, F., Caserini, S. & Renforth, P. Life cycle assessment of ocean liming for carbon dioxide removal from the atmosphere. J. Cleaner Prod. 370, 133309 (2022). https://doi.org/10.1016/j.jclepro.2022.133309
5. Marston, C., Rowland, C. S., O'Neil, A. W. & Morton, R. D. Land Cover Map 2021 (10 m classified pixels, Great Britain). NERC Environmental Information Data Centre (2022a). https://doi.org/10.5285/a22baa7c-5809-4a02-87e0-3cf87d4e223a
6. Marston, C., Rowland, C. S., O'Neil, A. W. & Morton, R. D. Land Cover Map 2021 (10 m classified pixels, Northern Ireland). NERC Environmental Information Data Centre (2022b). https://doi.org/10.5285/e44ae9bd-fa32-4aab-9524-fbb11d34a20a
