# Supplementary material for: Larger rock extraction sites could improve the efficiency of enhanced rock weathering in the United Kingdom
Source: Commun Earth Environ. 2025 Aug 15;6(1):666. doi: 10.1038/s43247-025-02656-9 (PMC12356697; doi:10.1038/s43247-025-02656-9)
Supplement: Supplementary file 3 — Description of Additional Supplementary Files [file 43247_2025_2656_MOESM3_ESM.pdf]

Description of Additional Supplementary Files

Supplementary Data 1

Source data for Figure 1. Potential of enhanced rock weathering in the UK 2025-2070.

Supplementary Data 2

Source data for Figure 3. Potential of enhanced rock weathering in the UK 2025-2070.

Supplementary Data 3

Source data for Figure 5. Transport requirement for ERW in the UK under the high rock supply scenario (S3.c).
